# A link between *Rel*B expression and tumor progression in laryngeal cancer

**DOI:** 10.18632/oncotarget.23109

**Published:** 2017-12-09

**Authors:** Ioanna Giopanou, Ioannis Lilis, Helen Papadaki, Theodoros Papadas, Georgios T. Stathopoulos

**Affiliations:** ^1^ Laboratory for Molecular Respiratory Carcinogenesis, Department of Physiology, Faculty of Medicine, University of Patras, Rio, Achaia 26504, Greece; ^2^ Department of Anatomy, Faculty of Medicine, University of Patras, Rio, Achaia 26504, Greece; ^3^ Department of Otorhinolaryngology & Head and Neck Surgery, Faculty of Medicine, University of Patras, Rio, Achaia 26504, Greece; ^4^ Comprehensive Pneumology Center (CPC) and Institute for Lung Biology and Disease (iLBD), University Hospital, Ludwig-Maximilians University and Helmholtz ZentrumMünchen, Member of The German Center for Lung Research (DZL), Munich, Bavaria 81377, Germany

**Keywords:** head and neck squamous cell carcinoma, nuclear factor (NF)-κB, biomarker, immunohistochemistry, prognosis

## Abstract

Laryngeal cancer is a frequent malignancy originating from the squamous vocal epithelium in a multi-stage fashion in response to environmental carcinogens. Although most cases can be cured by surgery and/or radiotherapy, advanced and relapsing disease is common, and biomarkers of such dismal cases are urgently needed. The cancer genome of laryngeal cancers was recently shown to feature a signature of aberrant nuclear factor (NF)-κB activation, but this finding has not been clinically exploited. We analyzed primary tumor samples of 96 well-documented and longitudinally followed patients covering the whole spectrum of laryngeal neoplasia, including 21 patients with benign laryngeal diseases, 15 patients with dysplasia, 43 patients with early-stage carcinoma, and 17 patients with locally advanced carcinoma, for immunoreactivity of *Rel*A, *Rel*B, P50, and P52/P100, the main NF-κB subunits that activate transcription. Results were cross-examined with indices of tumor progression and survival. Interestingly, *Rel*B expression increased with tumor stage, grade, and local extent. Moreover, patients displaying high *Rel*B immunoreactivity exhibited statistically significantly poorer survival compared with patients featuring low levels of *Rel*B expression (*P* = 0.018 by log-rank test). Using Cox regression analyses and tumor stage, local extent, grade and *Rel*A/*Rel*B immunoreactivity, we develop a new score that can independently predict survival of patients with laryngeal cancer. Hence we provide a simple and affordable NF-κB-based test to predict prognosis in laryngeal cancer.

## INTRODUCTION

Laryngeal cancer is the most frequent head and neck squamous cell carcinoma (HNSCC) with constantly decreasing five-year survival rates [1, 2]. Although surgery and radiotherapy provide for long-term survival, a large fraction of newly diagnosed patients display already advanced disease, while a significant proportion of treated patients will relapse post-therapy, culminating in half of the patients eventually succumbing to the disease [3]. Although the prospective identification of these patients that anticipate a poor prognosis is desirable, this currently relies on preoperative and surgical staging, while biomarkers of aggressive disease are unavailable, and risk factors implicated in the pathogenesis, such as tobacco smoking, alcohol consumption, asbestos, dietary factors, and viral infection are of limited clinical utility [4–8].

A recent multi-platform analysis of the comprehensive genomes of 279 HNSCCs that included 72 laryngeal carcinomas identified aberrant activation of nuclear factor (NF)-κΒ as critical in the pathogenesis and for the development of new therapies [9]. NF-κB is mainly activated by nuclear translocation and activation of transcription by one or more of four subunits: *Rel*A, *Rel*B, P50, and P52/P100. These ubiquitous proteins form cytoplasmic homo- or heterodimers bound to inhibitors of NF-κB (ΙκΒ). Upon stimulation, IκB undergo phosphorylation by multiple IκB kinases (IKK), as well as ubiquitination and proteolytic degradation by the proteasome and other proteases, and release active NF-κB subunits which translocate to the nucleus and activate transcription [10]. There are two main pathways leading to NF-κB activation. The canonical pathway is mainly mediated by *Rel*A/P50 and the non-canonical or alternative by *Rel*B/P52 dimers [10–12]. NF-κB activation occurs after stimulation of benign cells by cytokines, bacteria or viruses, endotoxins, oxidative stress, irradiation, etc, but can be constitutive in cancer cells [13, 14]. Oncogenic NF-κB activity has been documented in several human cancers [15–18] and has been shown to be functionally involved in HNSCC progression [19–22]. However, a simple and cheap NF-κB-based test clinically useful to identify laryngeal cancer patients that face a poor prognosis is missing.

We used simple immunohistochemistry to analyze the expression of the four main NF-κB subunits on serial sections of primary tumor samples from 96 well-documented and longitudinally followed patients spanning the whole spectrum of laryngeal neoplasia. A simple, fast, and reproducible NF-κB scoring system that examines immunoreactivity intensity, extent, and nuclear localization was employed [23]. We show how using this clinically relevant approach, *Rel*B expression was found to increase with tumor stage, grade, and local extent and to portend poor survival, establishing it as a useful biomarker of prognosis in laryngeal cancer.

## RESULTS

### A prospective cohort of laryngeal neoplasia

**Figure 1:**
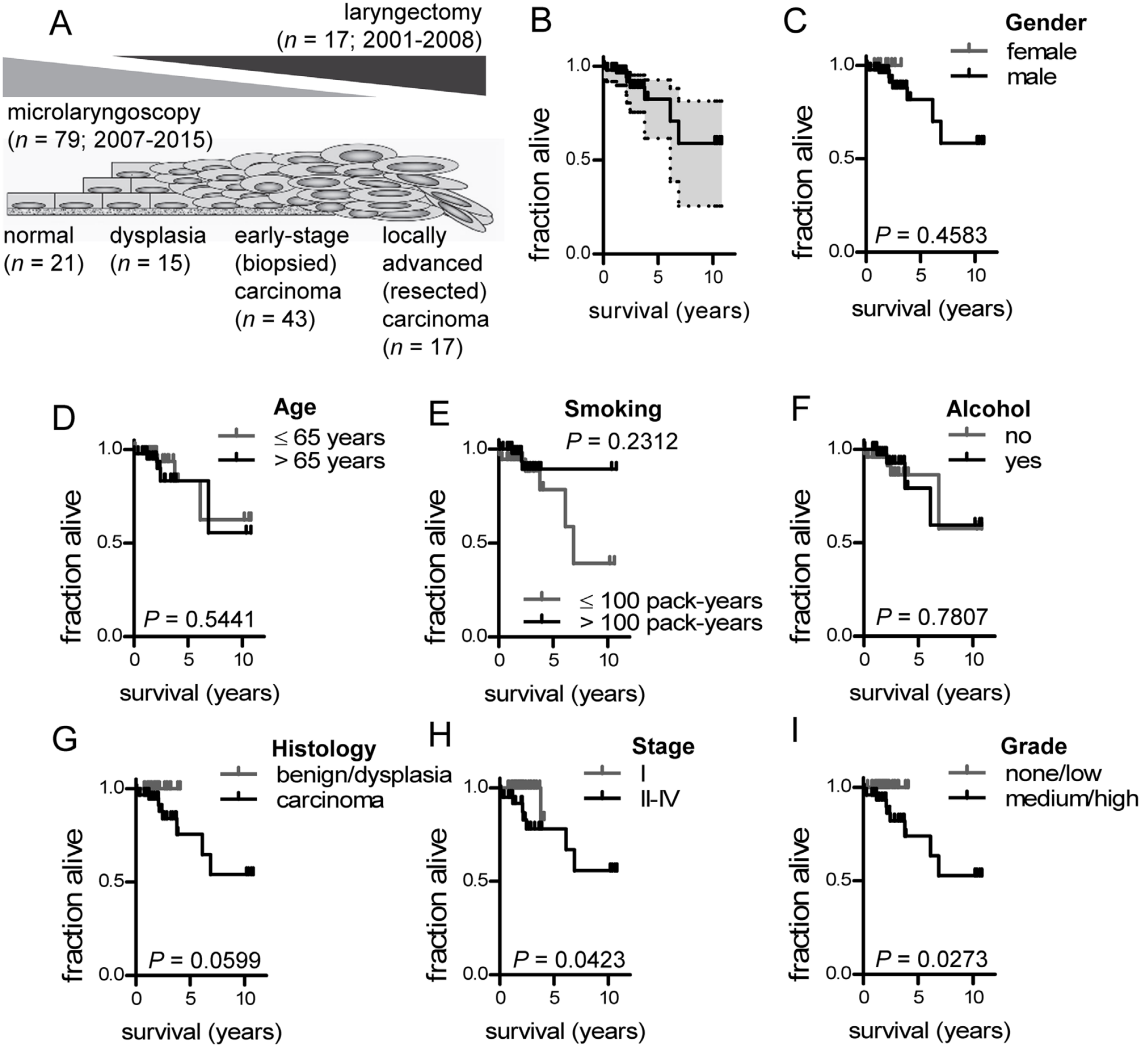
Study design and survival of 96 patients with benign and malignant laryngeal disease **(A)** Schematic representation of patient clinicopathologic categories and their distribution across the spectrum of laryngeal neoplasia. **(B)** Overall Kaplan-Meier survival plot with 95% confidence interval. **(C-I)** Kaplan-Meier survival estimates of patients stratified by gender (C; female: *n* = 10; male: *n* = 86), age (D; ≤65 years: *n*=49; >65 years: n=47), smoking (E; ≤100 pack years: *n*= 37; >100 pack years: *n=* 59), alcohol (F; no: *n*= 48; yes: *n*= 48), clinicopathologic category (G; benign/dysplasia: *n* = 36; carcinoma: *n* = 60), TNM7 stage (H; I: *n* = 55; II-IV: *n* = 41), and tumor grade (I; none/low: *n* = 47; medium/high: *n* = 49). *n*, sample size; *P*, probability by log-rank test.

Ninety-six patients were prospectively enrolled in the study. All were Caucasian, 86 were male, and median (interquartile range) follow-up was 24 (16-34) months. Twenty-one had benign laryngeal disease, 15 had dysplasia, 43 had early-stage carcinoma, and 17 had locally advanced carcinoma (Figure 1A). The clinical and pathologic features of the study patients are summarized in Table 1. Overall median survival was > 12 years (undefined by Kaplan-Meier analysis), while 94, 93, 79, and 57 patients survived to 1, 2, 5, and 10 years post-diagnosis, respectively (Figure 1B). There was no impact of gender, age, smoking, and alcohol intake on survival (Figure 1C-1F). As expected, patients with carcinoma, TNM7 stage II-IV disease, and medium/high grade histology displayed shorter survival (Figure 1G-1I).

**Table 1:** Clinical-pathologic features of 96 patients with laryngeal cancer

	Benign laryngeal disease	Dysplasia	Early-stage carcinoma	Locally advanced carcinoma
**Gender** (male/female; *n*)	13/8	15/0	42/1	16/1
**Age** (years; range)	24-75	48-76	53-90	49-88
**Smoking** (never/ex/current; *n*)	3/8/10	0/1/14	0/5/38	2/2/13
**Alcohol intake** (no/yes; *n*)	16/5	3/12	21/22	8/9
**Tumor grade** (*n*)				
Not specified	21	15	2	1
Low	0	0	4	4
Intermediate	0	0	26	5
High	0	0	11	7
**TNM7 stage**(*n*)				
Not applicable	21	15	0	0
I	0	0	18	1
II	9	9	11	2
III	0	0	10	8
IV	0	0	4	6

### Increased *Rel*B and P50 expression in advanced laryngeal cancer

**Figure 2:**
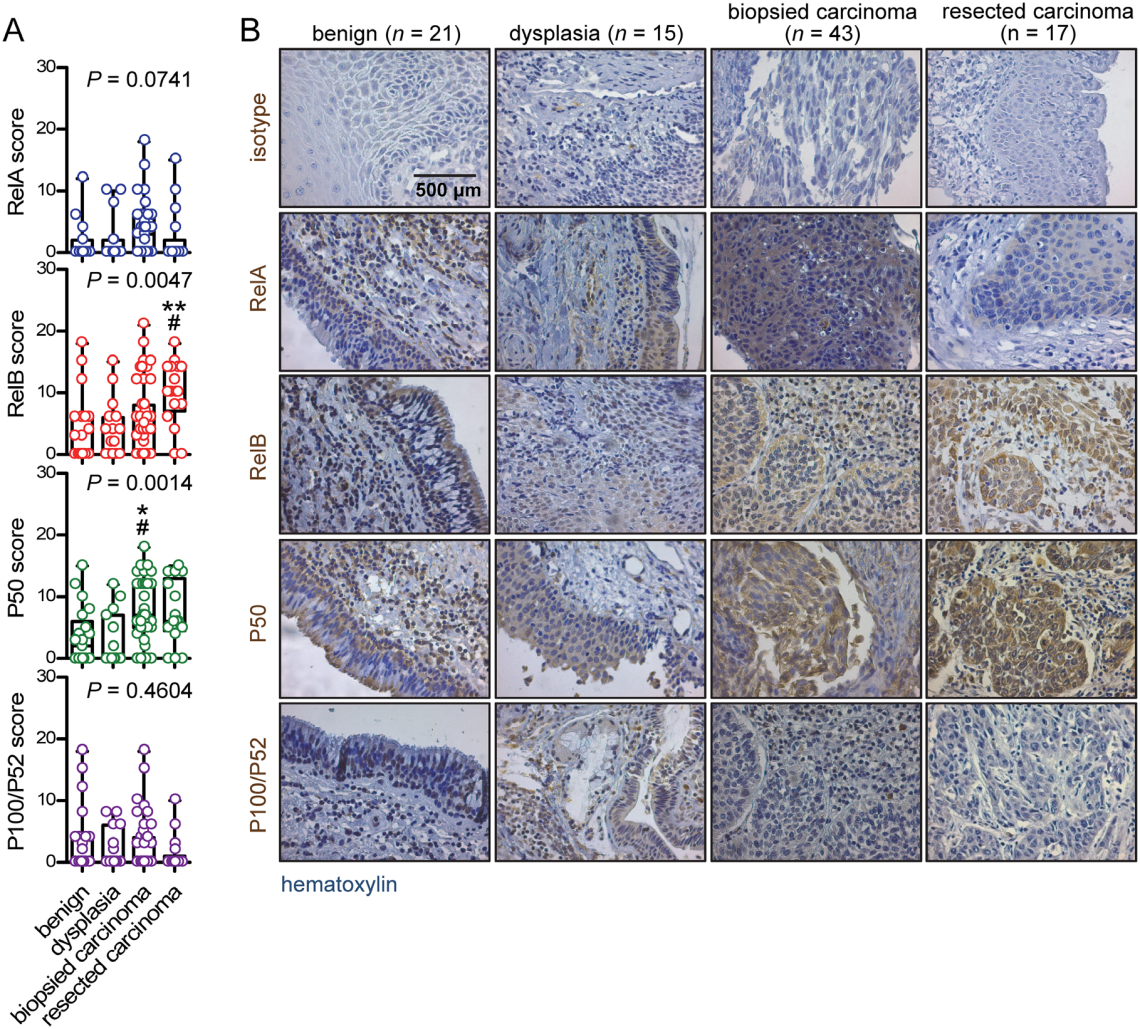
Immunohistochemical detection of NF-κB subunit expression by clinicopathologic study group **(A)** Data summary shown as raw data points and bars (median) with boxes (interquartile range) and whiskers (95% percentiles). **(B)** Representative images.*n*, sample size; *P*, overall probability by Kruskal-Wallis test. ^*^ and ^**^: *P*< 0.05 and *P*< 0.01, respectively, for comparison with benign group by Dunn’s post- tests. ^#^: *P*< 0.05 for comparison with dysplasia group by Dunn’s post-tests.

**Figure 3:**
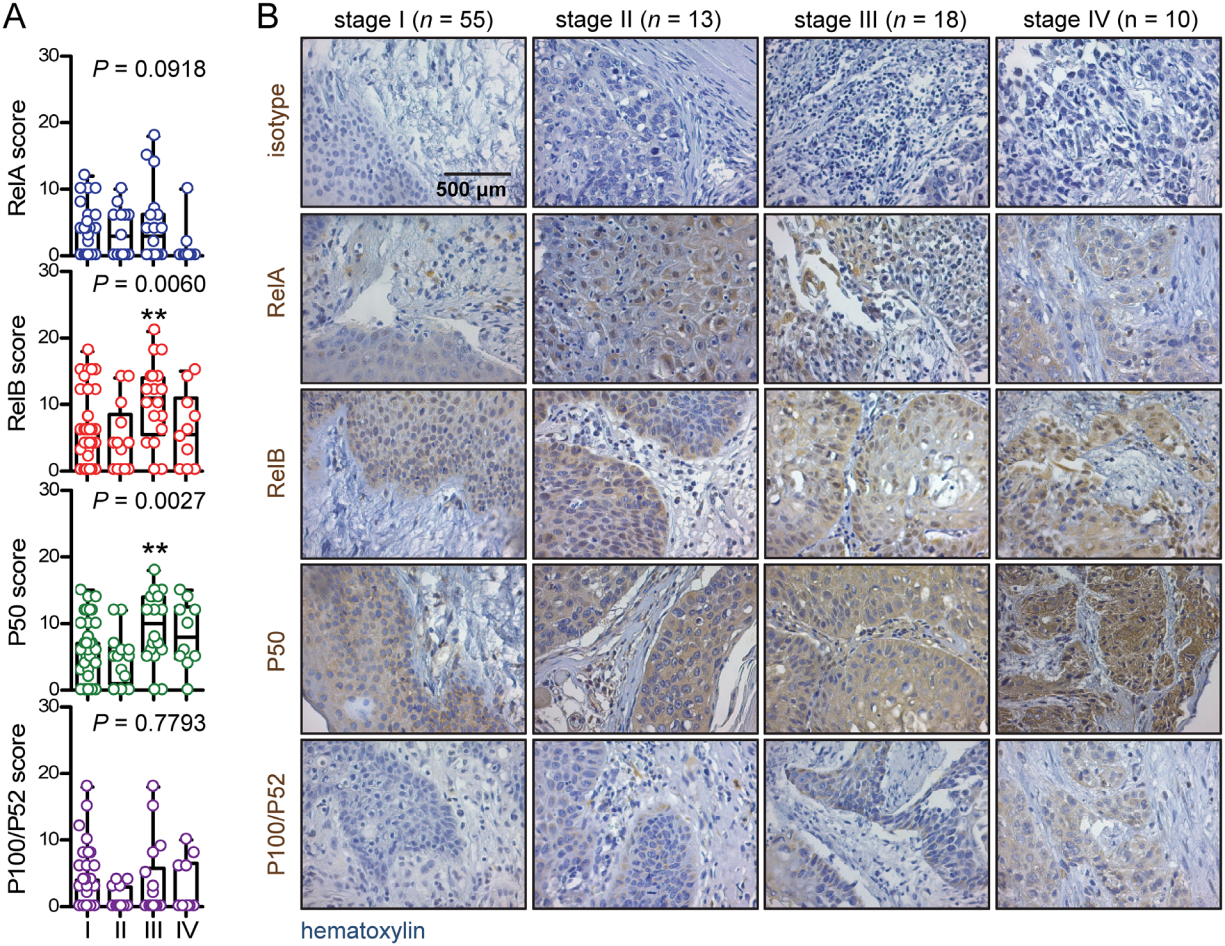
NF-κB subunit expression by TNM7 stage **(A)** Data summary shown as raw data points and bars (median) with boxes (interquartile range) and whiskers (95% percentiles). **(B)** Representative images.*n*, sample size; *P*, overall probability by Kruskal-Wallis test. ^**^: *P*< 0.01 for comparison with benign group by Dunn’s post-tests.

**Figure 4:**
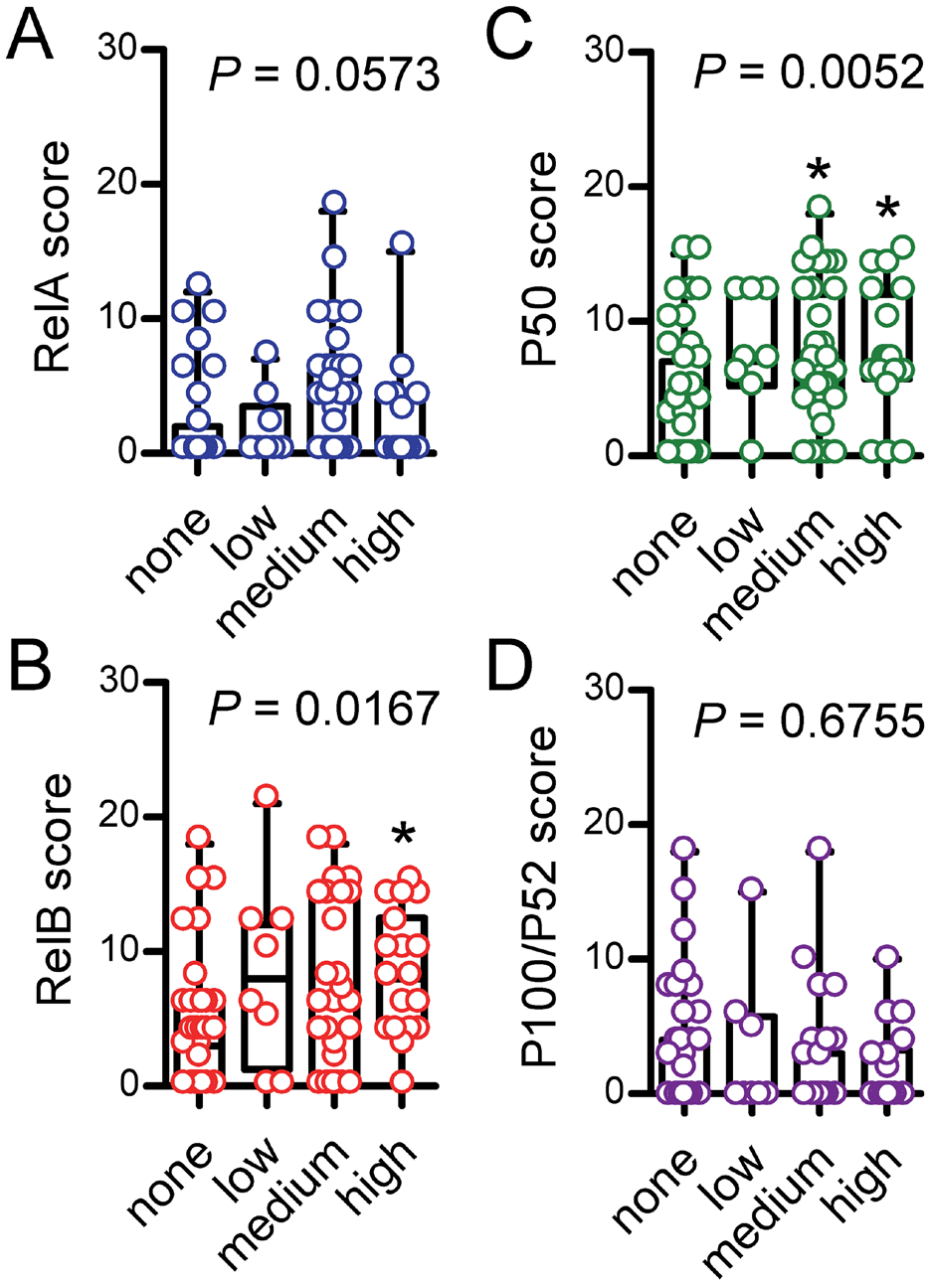
Immunohistochemical detection of NF-κB subunit expression by tumor grade Data summary shown as raw data points and bars (median) with boxes (interquartile range) and whiskers (95% percentiles). *n* = 39, 8, 31, and 18, respectively, for none (not applicable or specified), low, intermediate, and high grade groups. *P*, overall probability by Kruskal-Wallis test. ^*^: *P*< 0.05 for comparison with benign group by Dunn’s post-tests.

We next compared NF-κB subunit expression assessed by simple immunohistochemistry across our four study groups (clinicopathologic categories of benign laryngeal disease, dysplasia, biopsied carcinoma, and resected carcinoma). We found no significant differences in *Rel*A and P100/P52 expression, but P50 was significantly increased in biopsied carcinomas and *Rel*B in resected ones (Figure 2). A similar pattern was evident when patients were subgrouped according to TNM7 stage, with no differences being evident for *Rel*A and P100/P52 expression across tumor stages and with increased P50 and *Rel*B expression in stage III patients compared with stage I/II patients (Figure 3). NF-κB subunit expression was also examined in respect to tumor grade, revealing no changes in *Rel*A and P100/P52 expression, but enhanced P50 and *Rel*B expression with increasing tumor grade (Figure 4). These findings indicated that P50 and *Rel*B expression increase with laryngeal cancer progression, as determined by tumor stage, grade, and local extent.

### *Rel*B expression predicts survival in laryngeal cancer

**Figure 5:**
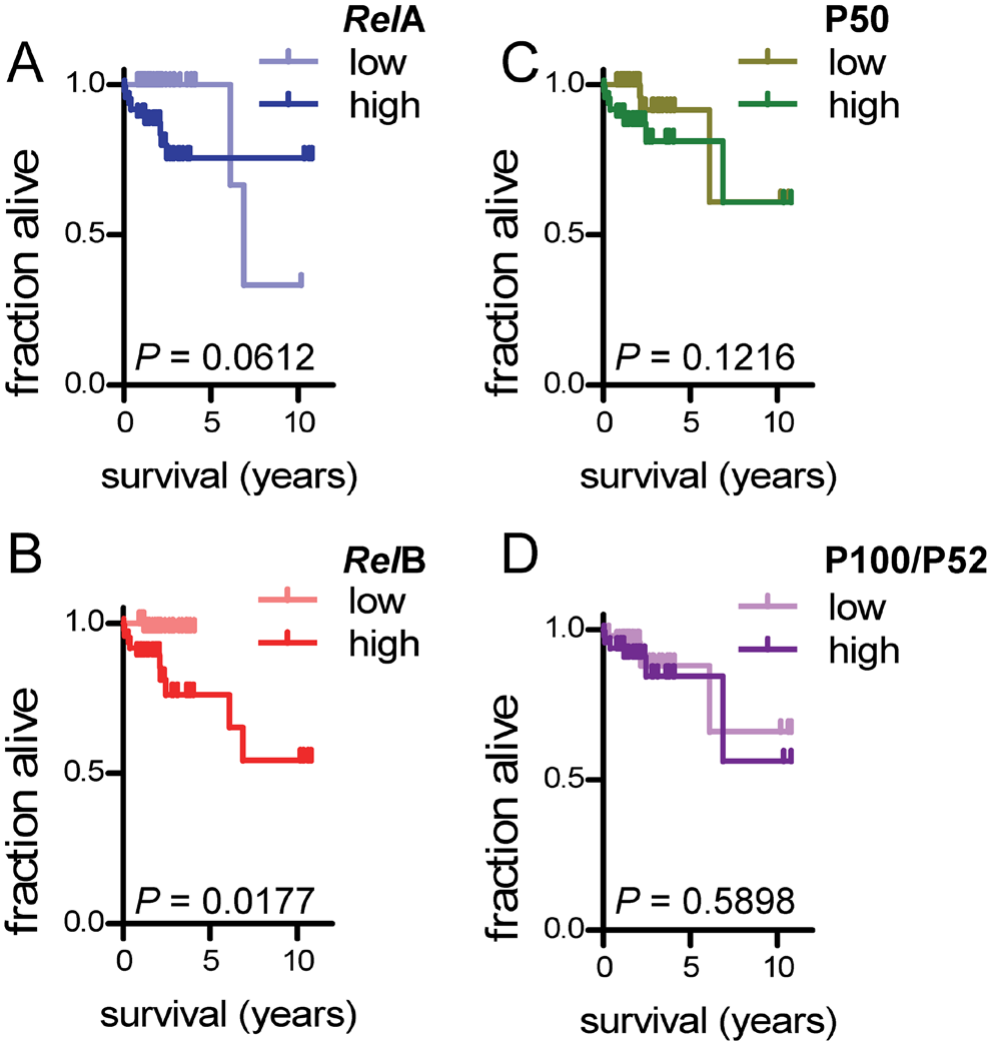
Survival by NF-κB subunit expression Shown are Kaplan-Meier survival estimates of patients dichotomized by median NF-κB subunit expression score (*n* = 48/group for all groups and graphs). *n*, sample size; *P*, probability by log-rank test.

**Figure 6:**
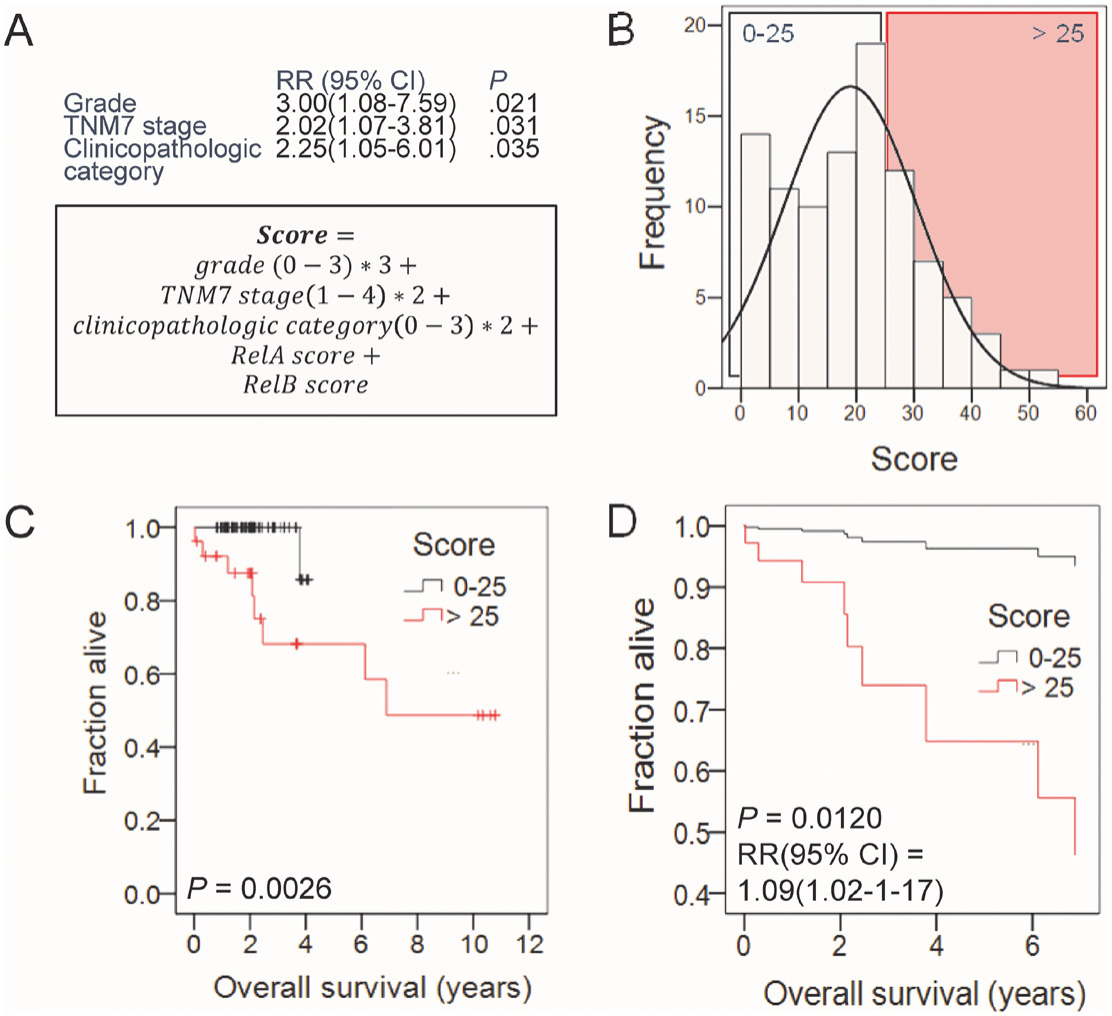
Cox regression analysis of the impact of clinical variables, risk factors, and NF-κB subunit expression on survival **(A)** Risk ratios (RR) with 95% confidence intervals (CI) and probability values (*P*) of the of the independent impact of the listed variables on survival. Note that successive variable only emerged as important after elimination of the preceeding variables. Equation showing the proposed laryngeal cancer prognostic score. **(B)** Frequency distribution of study cohort according to the newly devised score showing the two groupings with low (0-25; *n* = 70) and high (> 25; *n* = 26) scores. **(C)** Kaplan-Meier survival estimates of laryngeal cancer patients with low (0-25) and high (> 25) scores. *P*, probability by log-rank test. **(D)** Cox regression survival estimates of laryngeal cancer patients with low (0-25) and high (> 25) scores. *P*, probability by proportional hazards model. RR, Risk ratio of high versus low score. CI, 95% confidence interval.

In order to define a potential role for NF-κB subunit expression in predicting survival, we dichotomized our study cohort into low and high expression groups by the median value for each subunit (always *n* = 48/group). Interestingly, we found no significant differences in survival between patient groups expressing different levels of *Rel*A, P100/P52, and P50 (Figure 5A, 5C, 5D). However, patients with high *Rel*B expression displayed statistically significant shorter survival compared with patients with low *Rel*B expression (Figure 5B). We next entered all clinical variables and NF-κB subunit expression group (low or high for each different subunit) into Cox regression analysis using survival as the end-point and the Waldman backward elimination method (Figure 6). Tumor grade emerged as the only independent predictor of survival, suggesting that tumor stage, local extent necessitating intervention, and *Rel*B expression are interconnected predictors of survival in laryngeal cancer. When tumor grade was eliminated, TNM7 stage emerged as an independent predictor of survival, and when TNM7 stage was eliminated, clinicopathologic category significantly predicted survival (Figure 6A; Table). We extracted proportional hazards ratios from these analyses and combined them with *Rel*A and *Rel*B scores (which marginally and significantly predicted survival in Kaplan-Meier analyses shown in Figure 5) to form a proportional laryngeal cancer prognostic score (Figure 6A; equation). Based on the distribution of patients according to the new score, they were dichotomized using a cut-off of 25 (Figure 6B). The newly devised laryngeal cancer prognostic score accurately and significantly predicted survival on Kaplan-Meier and Cox proportional hazards analyses, indicating that NF-κB subunit expression cooperates with clinical grade, stage, and extent to define survival (Figure 6C and 6D).

### *Rel*B and P50 expression are linked with tumor progression in laryngeal cancer

**Figure 7:**
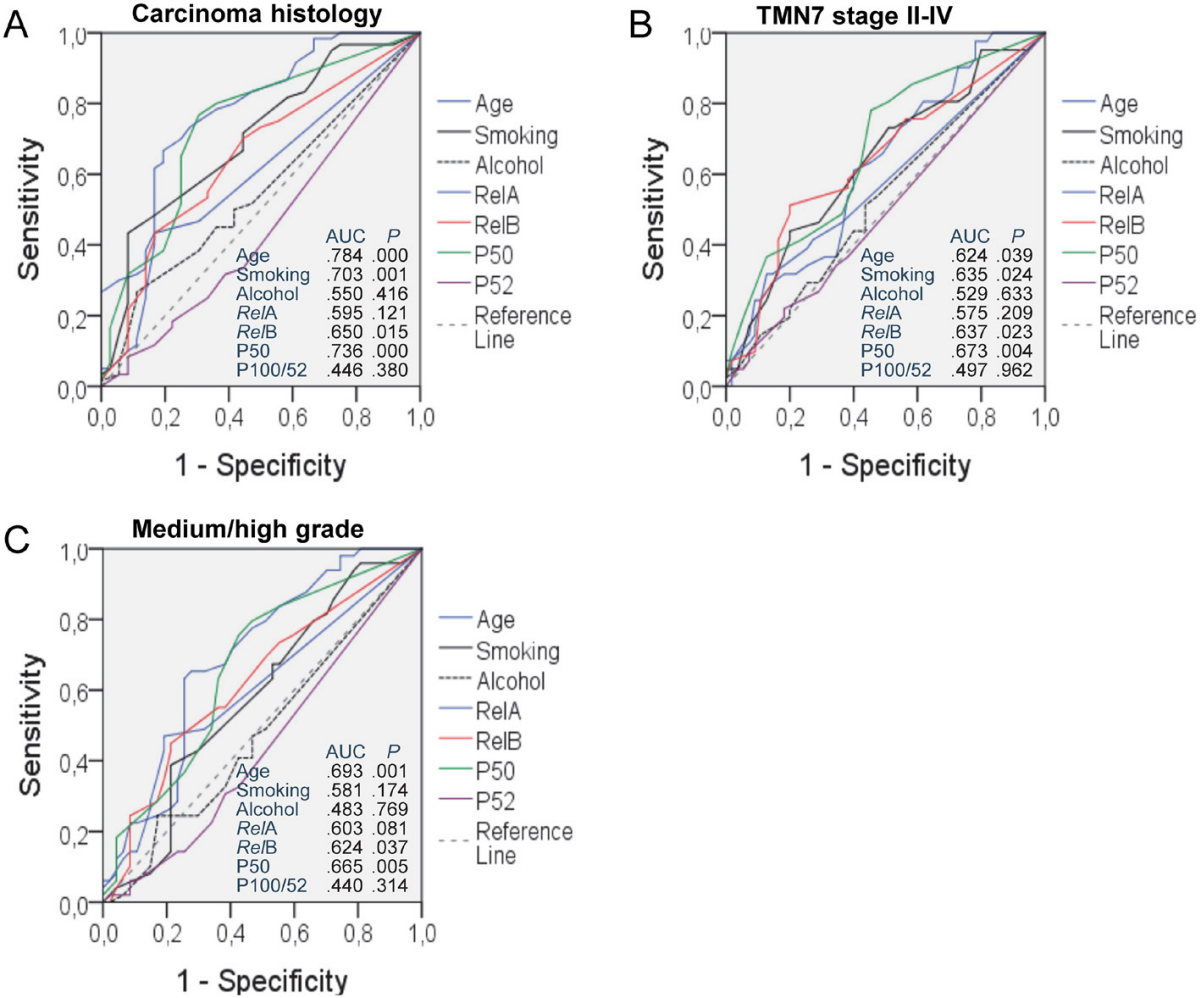
Receiver-operator curve (ROC) analysis of the impact of clinical variables, risk factors, and NF-κB subunit expression on tumor histology **(A)**, stage **(B)**, and progression **(C)**. AUC, area under curve; P, probability.

In order to better define the association between clinicopathologic variables and NF-κB expression with tumor progression, we performed ROC analyses. As shown in Figure 7, increasing age, smoking exposure, and *Rel*B and P50 expression were statistically significantly and positively associated with tumor extent, stage, and grade (Figure 7A-7C). However, we failed to identify such relationships for alcohol consumption and *Rel*A and P100/P52 expression (Figure 7A-7C).

## DISCUSSION

Based on two previous reports that identified the cardinal significance of NF-κB signaling in HNSCC, we characterized the expression and subcellular localization of the main NF-κB pathway effector proteins in human laryngeal cancer. We employed simple immunohistochemistry and an own-devised scoring system to achieve clinically relevant methods and results. We studied a carefully designed patient cohort reflecting the whole spectrum of progressive laryngeal neoplasia. Importantly, we followed patients for several years over a total study period of 18 years to obtain robust and valuable survival data for a cancer type that has a relatively good prognosis. Our simple but robust approach identifies that NF-κB subunits, especially P50 and *Rel*B are linked with laryngeal cancer progression. Moreover, that *Rel*B assessed at the time of diagnosis can predict the survival of patients with this cancer type.

The prognosis of patients with larynx cancer is currently assessed using the TNM7 staging system, while risk factors such as smoking and viral infection cannot accurately predict survival [9, 24]. However, TNM staging in the post-laryngectomy era is anatomic imaging based, labor-intensive, and not always accurate, as some early-stage tumors will relapse. To this end, a biologic marker of prognosis would be advantageous, especially one that is easily performed and quantified [25].

Although the importance of NF-κB signaling in HNSCC has been long suspected [26–29], it was recently independently established by a comprehensive study of 279 human HNSCC genomes and by a functional investigation of the two main NF-κB kinases [9, 19]. However, the findings of these two hallmark studies remained clinically unexploited. Here we show that *Rel*B expression assessed by immunohistochemistry and a simple scoring system at the time of diagnosis can be used as a cheap and accurate bedside test to predict the prognosis of patients with laryngeal cancer. Moreover, that *Rel*A and *Rel*B expression can be combined with clinical variables to form a prognostic score that robustly predicts survival.

Overexpression of *Rel*A and P50 has been previously reported in laryngeal carcinoma and has been linked to tumor progression, therapy response, and prognosis [26–31], but the potential role of *Rel*B as a biomarker of progression and survival is still under investigation. In non-small cell lung cancer, a tumor type with high similarity to HNSCC, two previous studies identified similar roles for *Rel*B as a potential biomarker of tumor progression and survival [23, 32]. In addition, a more recent study of head and neck squamous cell carcinoma proposed a combined effect of both IKKα and IKKβ on the nuclear localization of canonical *Rel*A and alternative *Rel*B and P100/P52 subunits [19]. This could provide a hint that alternative NF-κB activation stimulates altered intracellular and paracrine signaling from tumor cells, since *Rel*A-P50 and *Rel*B-P100/P52 complexes bind to NF-κB binding sites of different promoters [33]. The mechanism of the observed impact of *Rel*B is unknown and is worth to be explored by future studies. A possible explanation for the predominant effects of *Rel*B is nuclear membrane transporter chromosomal region maintenance/exportin1 protein (CRM1) that is linked with tumor progression in different types of cancers [34]. CRM1 is known to export *Rel*A from the nucleus, a mechanism that could explain the observed cytoplasmic localization of *Rel*A in our samples and that could render *Rel*B the only nuclear NF-κB transactivator in laryngeal and other cancers.

Identifying the link between P50 and *Rel*B with laryngeal cancer progression could be mechanistically explained by the fact that *Rel*B, which is known to form dimers with P100/P52, has been recently published to form heterodimers with P50 [35–36]. This phenomenon can also explain the findings of the present study, indicating that *Rel*B acts by forming canonical side-by -side heterodimers with P50. Our identification of *Rel*B activation is accompanied by the recent observation that the lymphotoxin-β receptor, whose activation results in alternative NF-κB activation, is overexpressed in a wide range of tumors [37]. Furthermore, NSCLC tumors, tightly linked to laryngeal cancer by cellular origin and histology, have been reported to express another activator of the alternative NF-κB pathway, CD40 and its ligand, CD154, providing another possible molecular mechanism for alternative NF-κB activation [38]. Additional reports identified that elevated nuclear *Rel*B in cancer cells promotes tumorigenicity and leads to elevated plasma interleukin-8 levels [39]. Consistent with our results, interleukin-8 is constitutively expressed in many metastatic cancers including HNSCC.

The proposed role for *Rel*B in laryngeal cancer is in line with other mechanistic studies that indicate it to be a promoter of oncogenic transcription and stemness in tumor initiating cells of colon cancers, gliomas, lymphomas, and myelomas [40–43]. Future studies designed to prospectively resample patients with laryngeal cancer after treatment could validate our findings and would assess the value of *Rel*B as a potential biomarker of treatment response. In addition to their clinical implications, our findings highlight the potential importance of non-canonical NF-κB signaling in cancer. Since most research has focused on the functions of components of the classical NF-κB activation pathway, such as P50 and *Rel*A, our findings, along with published functional studies [44, 45], underscore the necessity of further research into non-canonical NF-κB functions in the various cancer types.

In summary, our findings support that, pending further clinical validation, immunohistochemical assessment of *Rel*B expression at the time of diagnosis of laryngeal cancer is intimately linked with tumor progression and can accurately predict prognosis.

## MATERIALS AND METHODS

### Patients

Ninety-six patients who underwent microlaryngoscopy with diagnostic intent or laryngectomy with curative intent (before this technique was abandoned in 2008) between January 2001 and December 2015 at the University Hospital of Patras, Greece were prospectively enrolled in the study. The study’s observational protocol was conducted according to the Declaration of Helsinki, was approved by the Hospital Ethics Committee, and all patients gave written informed consent. Full clinical and pathologic data were recorded including age, gender, risk factors, histology, grade, and TNM7 stage. All patients were followed till death (actual events) or study conclusion (censored events) for overall survival. None of the patients received any anti-cancer drug treatment or irradiation before biopsy or laryngectomy, according to the best international clinical practice guidelines and recommendations. Multiple diagnostic tissue samples (3-5/patient) were formalin-fixed and paraffin-embedded and multiple hematoxylin and eosin-stained sections were evaluated by a certified pathologist (HP). Patients were classified into four clinicopathologic categories spanning the full spectrum of laryngeal neoplasia: benign (microlaryngoscopy-obtained benign nodules, chronic inflammation, polyps, metaplasia, etc.), dysplasia (microlaryngoscopy-obtained samples with mild, moderate, and severe dysplasia), early-stage carcinoma (microlaryngoscopy-obtained carcinoma biopsies), and locally advanced carcinoma (laryngectomy samples from patients with locally advanced carcinomas). All patients were staged according to the seventh edition of the American Joint Committee on Cancer TNM classification [24].

### Immunohistochemistry

Tissue blocks were cut into 4 μm-thick sections, placed onto polylysine-coated glass slides, deparaffinized by ethanol gradient, rehydrated, and boiled for 10 min in antigen retrieval solution (0.1 M sodium citrate; pH = 6.0). Endogenous peroxidase activity was inhibited using 3% H2O2 and non-specific antibody-protein binding was prevented using 3% bovine-serum albumin-containing Tris-buffered saline. The following primary antibodies and dilutions were used overnight at 4°C: anti-P50 (sc-114 rabbit polyclonal IgG; 1/150; Santa Cruz Biotechnology, Santa Cruz, CA), anti-P100/P52 (ab31409 rabbit polyclonal IgG; 1/150; Abcam, Cambridge, UK), anti-*Rel*A (sc-8008 mouse monoclonal IgG; 1/200; Santa Cruz), and anti-*Rel*B (sc-226 rabbit polyclonal IgG; 1/400; Santa Cruz). Detection of primary antibodies was performed using a horse radish peroxidase-conjugated polymer according to the manufacturer’s instructions (EnVision; Dako, Glostrup, Denmark) and diaminobenzidine as the chromogenic substrate. Sections were counterstained with hematoxylin, dehydrated, and mounted using Entellan (Merck Millipore, Darmstadt, Germany). For isotype controls, the primary antibody was omitted. Normal tonsil tissue was employed as positive control. Immunoreactivity was scored by three blinded investigators (IG, IL, and HP) and consensus was sought in ambiguous cases by co-observation. In order to have a more complete description of immunoreactivity for further statistical evaluation we first scored NF-κB subunit cytoplasmic and nuclear immunoreactivity separately. The intensity of cytoplasmic or nuclear immunoreactivity was scored as 0 for negative immunoreactivity, 1 for weak, 2 for moderate, and 3 for strong. The extent of cytoplasmic or nuclear immunoreactivity was scored as 0 for < 10% positive cells, 1 for 10-25% positive cells, 2 for 25-50% positive cells, 3 for 50-75% positive cells and 4 for > 75% positive cells. Nuclear distribution was scored as 0 when nuclear intensity ^*^ nuclear extent equaled 0; 1 for nuclear intensity ^*^ nuclear extent = 1-2; 2 for nuclear intensity ^*^ nuclear extent = 3-6 and 3 for nuclear intensity ^*^ nuclear extent = 6-16. Finally, the total NF-κB subunit score was calculated as (cytoplasmic intensity + cytoplasmic extent) ^*^ nuclear distribution. NF-κB subunit scores were further dichotomized into low and high expression by median values of the whole cohort. Images were taken using an upright AxioLab.A1 microscope connected to an AxioCamERc 5s camera (Zeiss, Jena, Germany).

### Statistics

The study was designed to include 112 patients across all four clinicopathologic patient categories in order to detect large biologic effect sizes (ρ = 0.4) with acceptable α and β errors of 0.05. However, *a posteriori* analyses including the 96 patients recruited at study conclusion indicated that statistical significance for primary end-points was reached with > 90% power. Power analyses were done using G^*^Power [46]. Survival analyses were done using Kaplan-Meier estimates and Log-rank (Mantel-Cox) tests. NF-κB subunit scores were not normally distributed, as tested by the Kolmogorov-Smirnov test (*P* < 0.05), and are presented as raw data points, bars (median) with boxes (interquartile range) and whiskers (95% percentiles). Matched scores for different NF-κB subunits of the same tumors were compared by Friedman’s test with Dunn’s post-tests. Unpaired scores of a given NF-κB subunit between groups of patients were compared by Kruskal-Wallis test with Dunn’s post-tests. Probability (P) values less than 0.05 were considered significant. Multivariate Cox regression survival analyses were done using backward (Waldman) elimination. Statistical analyses were performed using Prism v5.0.0 (GraphPad, San Diego, CA) and the Statistical Package for the Social Sciences v24 (IBM SPSS Statistics, Chicago, IL, USA).

## References

[1] Fitzmaurice C, Allen C, Barber RM, Barregard L, Bhutta ZA, Brenner H, Dicker DJ, Chimed-Orchir O, Dandona R, Dandona L, Fleming T, Forouzanfar MH, Hancock J (017). Global, regional, and national cancer incidence, mortality, years of life lost, years lived with disability, and disability-adjusted life-years for 32 cancer groups, 1990 to 2015: a systematic analysis for the global burden of disease study. JAMA Oncol.

[2] Chatenoud L, Garavello W, Pagan E, Bertuccio P, Gallus S, La Vecchia C, Negri E, Bosetti C (2016). Laryngeal cancer mortality trends in European countries. Int J Cancer.

[3] Groome PA, O’Sullivan B, Irish JC, Rothwell DM, Schulze K, Warde PR, Schneider KM, Mackenzie RG, Hodson DI, Hammond JA, Gulavita SP, Eapen LJ, Dixon PF (2003). Management and outcome differences in supraglottic cancer between Ontario, Canada, and the Surveillance, Epidemiology, and End Results areas of the United States. J ClinOncol.

[4] Peng WJ, Mi J, Jiang YH (2016). Asbestos exposure and laryngeal cancer mortality. Laryngoscope.

[5] Di Maso M, Talamini R, Bosetti C, Montella M, Zucchetto A, Libra M, Negri E, Levi F, La Vecchia C, Franceschi S, Serraino D, Polesel J (2013). Red meat and cancer risk in a network of case-control studies focusing on cooking practices. Ann Oncol.

[6] Sawabe M, Ito H, Oze I, Hosono S, Kawakita D, Tanaka H, Hasegawa Y, Murakami S, Matsuo K (2017). Heterogeneous impact of alcohol consumption according to treatment method on survival in head and neck cancer: a prospective study. Cancer Sci.

[7] Gama RR, Carvalho AL, Longatto Filho A, Scorsato AP, Lopez RV, Rautava J, Syrjanen S, Syrjanen K (2016). Detection of human papillomavirus in laryngeal squamous cell carcinoma: Systematic review and meta-analysis. Laryngoscope.

[8] Muscat JE, Liu HP, Livelsberger C, Richie JP, Stellman SD (2012). The nicotine dependence phenotype, time to first cigarette, and larynx cancer risk. Cancer Causes Control.

[9] Cancer Genome Atlas Network (2015). Comprehensive genomic characterization of head and neck squamous cell carcinomas. Nature.

[10] Gilmore TD (2006). Introduction to NF-kappaB: players, pathways, perspectives. Oncogene.

[11] Chen F, Castranova V, Shi X, Demers LM (1999). New insights into the role of nuclear factor-kappaB, a ubiquitous transcription factor in the initiation of diseases. Clin Chem.

[12] Sun SC (2011). Non-canonical NF-kappaB signaling pathway. Cell Res.

[13] Hayden MS, Ghosh S (2014). Regulation of NF-kappaB by TNF family cytokines. SeminImmunol.

[14] Oeckinghaus A, Ghosh S (2009). The NF-kappaB family of transcription factors and its regulation. Cold Spring HarbPerspect Biol.

[15] Cai Q, Tu M, Xu-Monette ZY, Sun R, Manyam GC, Xu X, Tzankov A, Hsi ED, Møller MB, Medeiros LJ, Ok CY, Young KH (2017). NF-κB p50 activation associated with immune dysregulation confers poorer survival for diffuse large B-cell lymphoma patients with wild-type p53. Mod Pathol.

[16] Smith SM, Lyu YL, Cai L (2014). NF-κB affects proliferation and invasiveness of breast cancer cells by regulating CD44 expression. PLoS One.

[17] Tang B, Tang F, Wang Z, Qi G, Liang X, Li B, Yuan S, Liu J, Yu S, He S (2016). Upregulation of Akt/NF-κB-regulated inflammation and Akt/Bad-related apoptosis signaling pathway involved in hepatic carcinoma process: suppression by carnosic acid nanoparticle. Int J Nanomedicine.

[18] Huang S, Pettaway CA, Uehara H, Bucana CD, Fidler IJ (2001). Blockade of NF-kappaB activity in human prostate cancer cells is associated with suppression of angiogenesis, invasion, and metastasis. Oncogene.

[19] Nottingham LK, Yan CH, Yang X, Si H, Coupar J, Bian Y, Cheng TF, Allen C, Arun P, Gius D, Dang L, Van Waes C, Chen Z (2014). Aberrant IKKalpha and IKKbeta cooperatively activate NF-kappaB and induce EGFR/AP1 signaling to promote survival and migration of head and neck cancer. Oncogene.

[20] Wang R, Guo Y, Ma H, Feng L, Wang Q, Chen X, Lian M, Wang H, Fang J (2016). Tumor necrosis factor superfamily member 13 is a novel biomarker for diagnosis and prognosis and promotes cancer cell proliferation in laryngeal squamous cell carcinoma. Tumour Biol.

[21] Zhang W, Liu Y, Wang CW (2014). S100A4 promotes squamous cell laryngeal cancer Hep-2 cell invasion via NF-kB/MMP-9 signal. Eur Rev Med Pharmacol Sci.

[22] Liu YL, Sun YN (2014). Down-regulation of testes-specific protease 50 induces apoptosis in human laryngocarcinoma HEp2 cells in a NF-κB-mediated pathway. MolBiol Rep.

[23] Giopanou I, Lilis I, Papaleonidopoulos V, Marazioti A, Spella M, Vreka M, Papadaki H, Stathopoulos GT (2015). Comprehensive evaluation of nuclear factor-kappabeta expression patterns in non-small cell lung cancer. PLoS One.

[24] Edge SB, Compton CC (2010). The American Joint Committee on Cancer: the 7th edition of the AJCC cancer staging manual and the future of TNM. Ann SurgOncol.

[25] Takes RP, Rinaldo A, Rodrigo JP, Devaney KO, Fagan JJ, Ferlito A (2008). Can biomarkers play a role in the decision about treatment of the clinically negative neck in patients with head and neck cancer?. Head Neck.

[26] Gupta A, Kumar R, Sahu V, Agnihotri V, Singh AP, Bhasker S, Dey S (2015). NFκB-p50 as a blood based protein marker for early diagnosis and prognosis of head and neck squamous cell carcinoma. BiochemBiophys Res Commun.

[27] Huang C, Huang K, Wang C, Jiang ZD, Li XX, Wang HP, Chen HY (2009). Overexpression of mitogen-activated protein kinase kinase 4 and nuclear factor-kappaB in laryngeal squamous cell carcinoma: a potential indicator for poor prognosis. Oncol Rep.

[28] Yamada T, Tsuda M, Wagatsuma T, Fujioka Y, Fujioka M, Satoh AO, Horiuchi K, Nishide S, Nanbo A, Totsuka Y, Haga H, Tanaka S, Shindoh M, Ohba Y (2016). Receptor activator of NF-κB ligand induces cell adhesion and integrin α2 expression via NF-κB in head and neck cancers. Sci Rep.

[29] Li Z, Yang Z, Passaniti A, Lapidus RG, Liu X, Cullen KJ, Dan HC (2016). A positive feedback loop involving EGFR/Akt/mTORC1 and IKK/NF-kB regulates head and neck squamous cell carcinoma proliferation. Oncotarget.

[30] Jiang LZ, Wang P, Deng B, Huang C, Tang WX, Lu HY, Chen HY (2011). Overexpression of Forkhead Box M1 transcription factor and nuclear factor-kappaB in laryngeal squamous cell carcinoma: a potential indicator for poor prognosis. Hum Pathol.

[31] Yoshida K, Sasaki R, Nishimura H, Okamoto Y, Suzuki Y, Kawabe T, Saito M, Otsuki N, Hayashi Y, Soejima T, Nibu K, Sugimura K (2010). Nuclear factor-kappaB expression as a novel marker of radioresistance in early-stage laryngeal cancer. Head Neck.

[32] Qin H, Zhou J, Zhou P, Xu J, Tang Z, Ma H, Guo F (2016). Prognostic significance of RelB overexpression in non-small cell lung cancer patients. Thorac Cancer.

[33] Fusco AJ, Huang DB, Miller D, Wang VY, Vu D, Ghosh G (2009). NF-kappaB p52: RelB heterodimer recognizes two classes of kappaB sites with two distinct modes. EMBO.

[34] Van der Watt PJ, Maske CP, Hendricks DT, Parker MI, Denny L, Govender D, Birrer MJ, Leaner VD (2009). The Karyopherin proteins, Crm1 and Karyopherin beta1, are overexpressed in cervical cancer and are critical for cancer cell survival and proliferation. Int J Cancer.

[35] Cormier F, Monjanel H, Fabre C, Billot K, Sapharikas E, Chereau F, Bordereaux D, Molina TJ, Avet-Loiseau H, Baud V (2013). Frequent engagement of RelB activation is critical for cell survival in multiple myeloma. PLoS One.

[36] Shih VF, Davis-Turak J, Macal M, Huang JQ, Ponomarenko J, Kearns JD, Yu T, Fagerlund R, Asagiri M, Zuniga EI, Hoffmann A (2012). Control of RelB during dendritic cell activation integrates canonical and noncanonical NF-κB pathways. Nat Immunol.

[37] Lukashev M, LePage D, Wilson C, Bailly V, Garber E, Lukashin A, Ngam-ek A, Zeng W, Allaire N, Perrin S, Xu X, Szeliga K, Wortham K (2006). Targeting the lymphotoxin-beta receptor with agonist antibodies as a potential cancer therapy. Cancer Res.

[38] Ishikawa K, Miyamoto M, Yoshioka T, Kato T, Kaji M, Ohbuchi T, Hirano S, Itoh T, Dosaka-Akita H, Kondo S (2008). Up-regulation of CD40 with juxtacrine activity in human nonsmall lung cancer cells correlates with poor prognosis. Cancer.

[39] Chan LP, Wang LF, Chiang FY, Lee KW, Kuo PL, Liang CH (2016). IL-8 promotes HNSCC progression on CXCR1/2-meidated NOD1/RIP2 signaling pathway. Oncotarget.

[40] Cha ST, Tan CT, Chang CC, Chu CY, Lee WJ, Lin BZ, Lin MT, Kuo ML (2016). G9a/RelB regulates self-renewal and function of colon-cancer-initiating cells by silencing Let-7b and activating the K-RAS/beta-catenin pathway. Nat Cell Biol.

[41] Roy P, Mukherjee T, Chatterjee B, Vijayaragavan B, Banoth B, Basak S (2017). Non-canonical NFκB mutations reinforce pro-survival TNF response in multiple myeloma through an autoregulatory RelB:p50 NFκB pathway. Oncogene.

[42] Ranuncolo SM, Pittaluga S, Evbuomwan MO, Jaffe ES, Lewis BA (2012). Hodgkin lymphoma requires stabilized NIK and constitutive RelB expression for survival. Blood.

[43] Lee DW, Ramakrishnan D, Valenta J, Parney IF, Bayless KJ, Sitcheran R (2013). The NF-kappaB*Rel*B protein is an oncogenic driver of mesenchymal glioma. PLoS One.

[44] Barbie DA, Tamayo P, Boehm JS, Kim SY, Moody SE, Dunn IF, Schinzel AC, Sandy P, Meylan E, Scholl C, Frohling S, Chan EM, Sos ML (2009). Systematic RNA interference reveals that oncogenic KRAS-driven cancers require TBK1. Nature.

[45] Seguin L, Kato S, Franovic A, Camargo MF, Lesperance J, Elliott KC, Yebra M, Mielgo A, Lowy AM, Husain H, Cascone T, Diao L, Wang J (2014). An integrin beta(3)-KRAS-RelB complex drives tumour stemness and resistance to EGFR inhibition. Nat Cell Biol.

[46] Faul F, Erdfelder E, Lang AG, Buchner A (2007). G^*^Power 3: a flexible statistical power analysis program for the social, behavioral, and biomedical sciences. Behav Res Methods.

